# Dehydration Status at Admission Predicts Recurrence in Patients with Traumatic Chronic Subdural Hematoma

**DOI:** 10.3390/jcm11051178

**Published:** 2022-02-22

**Authors:** Niklas Mainka, Valeri Borger, Alexis Hadjiathanasiou, Motaz Hamed, Anna-Laura Potthoff, Hartmut Vatter, Patrick Schuss, Matthias Schneider

**Affiliations:** 1 Department of Neurosurgery, University Hospital Bonn, 53127 Bonn, Germany; mainka_niklas@web.de (N.M.); valeri.borger@ukbonn.de (V.B.); alexis.hadjiathanasiou@ukb.de (A.H.); motaz.hamed@ukbonn.de (M.H.); anna-laura.potthoff@ukbonn.de (A.-L.P.); hartmut.vatter@ukbonn.de (H.V.); patrick.schuss@ukb.de (P.S.); 2 Department of Neurosurgery, BG Klinikum Unfallkrankenhaus Berlin, 12683 Berlin, Germany

**Keywords:** dehydration, chronic subdural hematoma, traumatic brain injury, recurrence

## Abstract

Objective: There remains a significant risk of chronic subdural hematoma (CSDH) recurring after treatment. Patient-related predictors and surgical procedures have been investigated in many studies. In contrast, the literature remains scant on reports of the potential impact of dehydration on the admission of affected patients and on the CSDH recurrence rate. Methods: All consecutively admitted patients with CSDH and surgical treatment at the authors’ institution between 2015 and 2019 were retrospectively identified. Dehydration was assessed as a blood urea/creatinine (U/Cr) ratio > 80. The association between dehydration on admission and postoperative complication rates, in-hospital mortality, and recurrence of CSDH, with the need for additional surgical treatment, was further analyzed. Results: A total of 265 patients with CSDH requiring surgery were identified. In 32 patients (12%), further surgery was necessary due to the recurrence of CSDH. It was found that 9 of the 265 patients with CSDH (3%) suffered from dehydration at the time of admission. Multivariate analysis revealed diabetes mellitus (*p* = 0.02, OR 2.7, 95% CI 1.2–6.5), a preoperative midline shift > 5 mm (*p* = 0.003, OR 3.3, 95% CI 1.5–7.5) and dehydration on admission (*p* = 0.002, OR 10.3, 95% CI 2.4–44.1) as significant and independent predictors for the development of CSDH recurrence that requires surgery. Conclusion: the present findings indicate that dehydration on admission appears to be an independent predictor for CSDH recurrence that requires surgery.

## 1. Introduction

Chronic subdural hematoma (CSDH) is a common sequel to mild traumatic brain injury (TBI) [1]. As a result, an accumulation of blood ensues in the subdural space, which subsequently becomes liquefied over time. Due to the aging population and associated use of anticoagulant medications, the incidence of CSDH is increasing to the point that surgical management of CSDH is predicted to be the most common cranial neurosurgical procedure in adults in the near future. Surgical evacuation of the subdural collection remains the main treatment approach for symptomatic patients [2].

However, up to 20% of patients suffer a recurrence of CSDH [3]. The prediction and, thus, prevention of such a frequently treatable recurrence of bleeding have been the aim of numerous scientific efforts. In addition to the size of the subdural hematoma, septation, age, pre-existing medical conditions, anticoagulant medications, and the placement/location of subdural drains are also enumerated as potential predictors of recurrent CSDH. The conventional manifestations of symptomatic CSDH are often cognitive impairment, gait disturbance, limb weakness and/or headache [1,2].

There is evidence that chronic mild dehydration constitutes a common condition in elderly individuals [4]. Even short periods of fluid restrictions might lead to a 1–2% reduction in body mass, culminating in increases in self-reported tiredness and headaches, as well as an accelerated decline in the already reduced thirst drives [5]. Moreover, dehydration constitutes a major determinant of morbidity and mortality in elderly patients [6].

In the commonly affected elderly population, such symptoms can often lead to lethargy, and, thus, an accelerated decline in the already reduced thirst drives. The eventual resulting dehydration constitutes a major determinant of morbidity and mortality in elderly patients [6]. Low habitual fluid intake has been linked to several chronic diseases, including urolithiasis, constipation, asthma, cardiovascular disease, elevated risk of infection and diabetic hyperglycemia [4,7,8]. The maintenance, secondary growth, and recurrence of CSDH are known to be driven by progressive inflammatory responses, which lead to intermittent bleeding from the outer CSDH membranes [9]. These inflammatory processes are reflected by mediators such as tissue plasminogen activator (tPA), thrombomodulin, angiopoietin-2, vascular endothelial growth factor and matrix metalloproteases [9,10,11,12,13]. Dehydration, in turn, has been reported to activate inflammatory signaling, increase the adhesive properties of endothelial cells and lead to vascular changes that promote thickening of the artery walls [14]. Therefore, dehydration-mediated systemic inflammatory responses might lead to the recruitment of immune cells, such as macrophages, that culminate in the reoccurrence of CSDH via the secretion of inflammatory mediators and reformation of inflammation-driven membranes in the subdural space. Against this background, we speculated whether an initial dehydration status might have an impact on CSDH recurrence in patients who have undergone surgical therapy for CSDH.

## 2. Materials and Methods

### 2.1. Patients

All medical records of consecutive patients with traumatic brain injury and CSDH treated at our department between 2015 and 2019 were retrospectively screened. Corresponding approval to conduct this study was granted after a detailed evaluation by the local ethics committee. Subsequently, clinical data on patient characteristics, clinical symptoms, comorbidities, initial laboratory values, Markwalder grading scale at admission, Glasgow coma scale (GCS) at admission, Glasgow outcome scale (GOS), modified ranking scale (mRS), and use of anticoagulant medications were extracted from the patient documentation. The diagnosis of CSDH was confirmed preoperatively by cranial computed tomography (CT). Using CT scans, CSDH was radiologically defined as subdural fluid collection of hypodense appearance (<25 Hounsfield units (HU)) compared to physiological brain parenchyma, homogeneous isodense appearance (25–35 HU), layered density type as an indication of several bleeding episodes, as well as mixed density type according to previously published literature [15]. Any anticoagulant medication was paused preoperatively and, depending on the urgency of the surgical procedure, active substitution was initiated according to internal standard operating procedures. Neurosurgical treatment was conducted under general anesthesia. The initial surgical procedure included a single burr hole craniotomy (BHC), and, in some cases, a craniotomy following a patient-specific decision made by the attending surgeon. Chronified subdural blood was aspirated by repeated irrigation with physiological saline. Subdural drainage with a closed system was then performed for several days. The duration of postoperative subdural drainage depended on the amount of subdural fluid/brain re-expansion, which was verified by a postoperative CT scan. Clinical and radiological follow-up by CT scan was obtained in all patients within 3 weeks after discharge from our neurosurgical outpatient department. If subdural fluid persisted, patients were followed up in the outpatient department until its resolution. After abatement of residual hematoma and absence of clinical consequences, any suspended anticoagulant medication was resumed under clinical supervision.

Recurrence of CSDH was defined as clinical recurrence with the onset of new neurologic symptoms, in combination with an increase in subdural fluid collection with compression of the brain surface, by comparing CT scans before and after surgery.

### 2.2. Definition of Dehydration

Currently, there is no standard for the clinical diagnosis of dehydration [16]. Thus, to assess the hydration status of patients with CSDH requiring surgery, serum urea (U) and serum creatinine (Cr) values at the time of preoperative hospitalization were extracted from patient records and used for further analysis. Here, the ratio between U and Cr was calculated for the estimation of dehydration [17]. As in previous reports, dehydration was then defined as a U/Cr ratio > 80, and an initial U/Cr ratio ≤ 80 was defined as the subgroup that was not dehydrated [18]. Furthermore, all available biomarkers of dehydration were expressed as binary variables. For urea, this was ≤7.5 and >7.5 mmol/L; for sodium (Na) ≤145 and >145 mmol/L; for estimated glomerular filtration rate (eGFR) <30 and ≥30 mL/min.

### 2.3. Statistics

All statistical analyses were conducted using the SPSS computer software package (version 25, IBMCorp., Armonk, NY, USA). Fisher’s exact test was utilized for the comparison of unpaired categorical and binary variables of the two groups with/without dehydration at the time of hospitalization. For the comparison of continuous variables, the Mann–Whitney U test was chosen since the data were mostly not normally distributed. Results with *p* < 0.05 were considered statistically significant.

In addition, a binary logistic regression analysis was performed to identify independent preoperative determinable predictors of recurrence of CSDH in patients who had previously undergone surgical therapy regarding the latter. With regard to the only 9 patients with preoperative dehydration, based on the available data, there was no statistically significant difference in variables that might differ in reality between the two groups of patients with or without dehydration. Therefore, the mere inclusion of variables in the multivariate analysis that reach statistical significance in the present univariate analysis may cause a loss of possibly valuable information regarding the prediction of CSDH recurrence, and was avoided in accordance with Heinze et al. [19]. This led to an additional variable selection based on clinical expertise.

## 3. Results

### 3.1. Patient Characteristics

Two hundred and sixty-five patients with CSDH underwent surgery at the Department of Neurosurgery, University Hospital Bonn, from 2015 to 2019. The median age of the treated patients was 79 years (interquartile range [IQR] 73–85). Ninety-five of the patients (36%) were female. Anticoagulant medication was present in 192 patients with CSDH (72%). In terms of pre-existing conditions, 53 patients (20%) suffered from diabetes mellitus and 160 patients (60%) from treatment-requiring arterial hypertension. The median preoperative Markwalder grading scale for the entire group of patients with CSDH was one (IQR 1–2). The respective value for GCS was 15 (14–15).

In 73% of cases, CSDH was unilateral (194/265). Overall, 218 patients (82%) with CSDH demonstrated some form of preoperative midline shift (MLS). In 99 patients (37%), MLS exceeded 5 mm. In 97 cases (37%), the presence of CSDH septations on the targeted side was noted on preoperative imaging. In 259 patients (98%), surgery consisted of BHC, whereas craniotomy was performed in 6 patients with CSDH (2%). The median operation time was 33 min (IQR 24–45). The median hospital stay amounted to 6 days (IQR 4–9).

### 3.2. Dehydration Status at Admission

Of the 265 patients with CSDH and surgical treatment in the present study, a total of 9 patients (3.4%) were dehydrated during their initial blood test performed on admission (U/Cr ratio > 80). The baseline characteristics of the patients with versus (vs.) without dehydration are detailed in Table 1. There were no significant differences in demographic characteristics, location/configuration and/or treatment of CSDH during hospitalization.

### 3.3. Risk of CSDH Recurrence

Overall, 32 patients (12%) suffered a recurrence of CSDH requiring surgery. In all the patients, recurrent CSDH was treated with revision surgery. The median GOS at discharge for the entire group of patients with CSDH was five (IQR 4–5). The respective value for mRS was one (0–3). The patients who had a recurrence of CSDH demonstrated a preoperative MLS > 5 mm significantly more often compared to patients without a CSDH recurrence (63% vs. 34%, *p* = 0.003, OR 3.2, 95% CI 1.5–6.9) (Table 2).

Neither pre-existing disease, unilateral/bilateral location of CSDH, nor the presence of septations in the hematoma differed significantly between the patients with and without a recurrence of CSDH. Pre-existing diabetes mellitus, which has been reported to constitute a risk factor for CSDH recurrence [20], occurred more frequently in the patients with subsequent CSDH recurrence than in the patients without recurrence, although no statistical significance was reached in this regard (*p* = 0.06).

### 3.4. Multivariate Analysis

**Figure 1:**
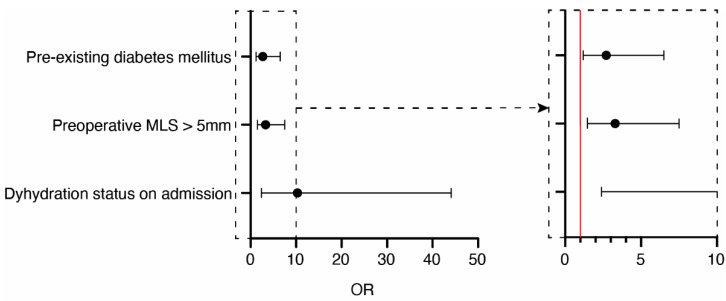
Forest plot. Visualization of the multivariate analysis for CSDH recurrence after initial surgical removal.

To identify the independent predictive factors for the post- surgery recurrence of CSDH, a multivariate analysis was conducted based on the dehydration status at admission, and preoperative MLS > 5 mm that had been identified within the univariate analysis, as well as diabetes mellitus, as a known and previously published risk factor [20]. The multivariate analysis revealed pre-existing diabetes mellitus (*p* = 0.02, OR 2.7, 95% CI 1.2–6.5), preoperative MLS > 5 mm (*p* = 0.003, OR 3.3, 95% CI 1.5–7.5), and dehydration status on admission (*p* = 0.002, OR 10.3, 95% CI 2.4–44.1) as significant and independent predictors for the recurrence of CSDH (Nagelkerkes R^2^ 0.164) (Figure 1).

## 4. Discussion

The results of the present series indicate that intake dehydration, detected by U/Cr > 80, might facilitate the recurrence of CSDH. Certainly, numerous risk factors for CSDH recurrence have previously been demonstrated. To begin with, there is the degree of septation within the hematoma itself [21,22]. Moreover, the benefits and risks of surgical approaches to individual membranes have been sufficiently and well explored/discussed in the past [23,24]. In addition to the characteristics of the hematoma (acute components) and septation, the manner of intraoperative irrigation, as well as insertion of subdural drainage, have also been discussed, in relation to the likelihood of CSDH recurrence [25,26,27,28]. Since this certainly constitutes a condition of the elderly, anticoagulant medications have also been mentioned as a potential risk factor for the recurrence of CSDH [29]. This circumstance will certainly be examined more meticulously in the future, since particularly high-risk patients, e.g., those with recent cardiological/endovascular interventions, are also dependent on anticoagulation during care for CSDH, which necessitates weighing up the risks and benefits of anticoagulant medications. Furthermore, the mere age of the patient is also discussed repeatedly as a possible risk factor for CSDH recurrence [29,30,31]. One line of argumentation is the decrease in brain volume in the elderly, leading to a larger extracerebral volume, which, in turn, may promote the development and progression of CSDH or its recurrence [32,33]. In addition, Jang et al. reported that brain volume depressed by the existing hematoma may also foster recurrence of CSDH, despite surgical evacuation [34]. A reduction in brain volume has also been reported to occur when patients are dehydrated [35], therefore constituting a direct focal (intracranial) correlation between dehydration status and CSDH recurrence. In addition, dehydration is known to induce inflammatory responses and increased adhesive properties in endothelial cells [14]. With regard to inflammatory mediators that facilitate secondary bleeding from CSDH membrane remnants after surgical therapy [9], dehydration might also impact CSDH recurrence in a systemic manner.

Dehydration is an important facilitator of morbidity and mortality, especially in older and, thus, often more vulnerable patients [36,37]. When an additional stressor (herein CSDH) compounds neurologic/cognitive impairment in already physically debilitated elderly patients, the impact of potential dehydration is further exacerbated. Nursing home patients, with additional dehydration, face additional poor outcomes [38]. Furthermore, a dehydrated state at the time of admission has also been found to significantly affect short-term outcomes in patients with acute ischemic stroke [18,39]. Marginal brain volume reduction, triggered by dehydration of the affected patient, can be partially compensated by rehydration, according to radiological studies [35]. The reciprocal effects of hypervolemic fluid management have also been recognized in the treatment and management of patients with acute brain injuries (e.g., subarachnoid hemorrhage). Montano et al. addressed the consideration of postoperative forced fluid therapy by providing saline solution intravenously to the treated CSDH patient until postoperative day 3 [40]. In this way, an additional reduction in the size of the CSDH was achieved—most likely by re-expansion of the brain. Janowski et al. had previously demonstrated a similar effect [41].

Since the present study demonstrated, for the first time, the impact of apparent prehospital dehydration in patients with CSDH on the likelihood of recurrence, it would be desirable for this to result in earlier identification of those patients who might benefit from such forced fluid management.

## 5. Limitations

This retrospective analysis, from only one medical facility, bears several shortcomings. The number of patients who actually suffered from significant dehydration is relatively small in the present patient cohort; therefore, the generalizability of the findings is limited. The authors intended to assess these data as the first estimation of a potential correlation between an initial dehydration status and the risk of postoperative CSDH recurrence, which might enable the initiation of further multicenter studies in order to cope with the limitation of potential selection bias due to the limited group size in the present study. The lack of a uniform definition of dehydration and the varying availability of all laboratory values make a thorough patient cohort presentation difficult. Furthermore, the retrospective nature did not allow for a causal analysis of reduced GFR values in the dehydration cohort, leaving mutual dependencies between these two variables unexplored. Nevertheless, this is the first attempt to demonstrate the impact of the dehydration status of affected patients with CSDH on the likelihood of recurrence, and should, therefore, be considered in future scientific endeavors.

## 6. Conclusions

The present findings indicate that dehydration on admission appears to be an independent predictor for CSDH recurrence requiring surgery.

## Figures and Tables

**Table 1:** Baseline characteristics of patients with and without dehydration.

Variables	Non-Dehydration (*n* = 256)	Dehydration (*n* = 9)	*p*-Value
**Median age (IQR, yrs)**	79 (73–85)	81 (74–86)	0.71
**Female sex**	91 (36%)	4 (44%)	0.73
**Preoperative anticoagulant medication**	186 (73%)	6 (67%)	0.32
Platelet inhibition	23 (9%)	2 (22%)	0.2
Vitamin K antagonists	123 (48%)	3 (33%)	0.5
Combination	40 (16%)	1 (11%)	1.0
**Median Markwalder grading scale (IQR)**	1 (1–2)	1 (0–1)	0.07
**Median GCS (IQR)**	15 (14–15)	15 (15–15)	0.55
**Unilateral CSDH**	185 (72%)	9 (100%)	0.12
**Preoperative MLS > 5 mm**	94 (37%)	5 (56%)	0.30
**Presence of CSDH septations**	91 (36%)	6 (67%)	0.08
**BHC**	250 (98%)	9 (100%)	0.82
**Admission Na > 145 mmol/L**	7 (3%)	1 (11%)	0.24
**Admission eGFR < 30 mL/min**	6 (2%)	9 (100%)	<0.0001
**Admission urea > 7.5 mmol/L**	58 (23%)	6 (67%)	0.007
**Recurrence of SDH**	27 (11%)	5 (56%)	0.002
**Median GOS at discharge (IQR)**	5 (4–5)	5 (4–5)	0.55
**Median mRS at discharge (IQR)**	1 (0–3)	0 (0–1)	0.1

**Table 2:** Baseline characteristics of patients with and without recurrence.

Variables	No CSDH Recurrence (*n* = 233)	CSDH Recurrence (*n* = 32)	*p*-Value
**Median age (IQR, yrs)**	79 (73–85)	80 (72–85)	0.68
**Female sex**	85 (37%)	10 (31%)	0.70
**Pre-existing diabetes mellitus**	42 (18%)	11 (34%)	0.06
**Pre-existing arterial hypertonus**	140 (60%)	20 (63%)	0.85
**Preoperative anticoagulant** **medication**	171 (73%)	21 (66%)	0.40
Platelet inhibition	22 (9%)	3 (9%)	1.00
Vitamin K antagonists	112 (48%)	14 (41%)	0.71
Combination	37 (16%)	4 (13%)	0.80
**Median Markwalder grading scale (IQR)**	1 (1–2)	1 (0–2)	0.83
**Median GCS (IQR)**	15 (14–15)	15 (14–15)	0.31
**Admission U/Cr > 80**	4 (2%)	5 (16%)	0.002
**Unilateral CSDH**	171 (73%)	23 (72%)	0.83
**Preoperative MLS > 5 mm**	79 (34%)	20 (63%)	0.003
**Presence of CSDH septations**	83 (36%)	14 (44%)	0.44
**BHC** **Length of hospital stay (IQR, days)**	228 (98%) 6 (4–9)	31 (97%) 6 (4–10)	0.54 0.86
**Median GOS at discharge (IQR)**	5 (4–5)	5 (4–5)	0.70
**Median mRS at discharge (IQR)**	1 (0–3)	1 (0–3)	0.77

## Data Availability

The data supporting the findings of this study are included within the article.

## References

[1] Kolias A.G., Chari A., Santarius T., Hutchinson P.J. (2014). Chronic subdural haematoma: Modern management and emerging therapies. Nat. Rev. Neurol..

[2] Gazzeri R., Laszlo A., Faiola A., Colangeli M., Comberiati A., Bolognini A., Callovini G. (2020). Clinical investigation of chronic subdural hematoma: Relationship between surgical approach, drainage location, use of antithrombotic drugs and postoperative recurrence. Clin. Neurol. Neurosurg..

[3] Cofano F., Pesce A., Vercelli G., Mammi M., Massara A., Minardi M., Palmieri M., D’Andrea G., Fronda C., Lanotte M.M. (2020). Risk of Recurrence of Chronic Subdural Hematomas After Surgery: A Multicenter Observational Cohort Study. Front. Neurol..

[4] Maughan R.J. (2012). Hydration, morbidity, and mortality in vulnerable populations. Nutr. Rev..

[5] Shirreffs S.M. (2003). Markers of hydration status. Eur. J. Clin. Nutr..

[6] Stookey J.D., Purser J.L., Pieper C.F., Cohen H.J. (2004). Plasma hypertonicity: Another marker of frailty?. J. Am. Geriatr. Soc..

[7] Manz F., Wentz A. (2005). The importance of good hydration for the prevention of chronic diseases. Nutr. Rev..

[8] Ferry M. (2005). Strategies for ensuring good hydration in the elderly. Nutr. Rev..

[9] Edlmann E., Giorgi-Coll S., Whitfield P.C., Carpenter K.L.H., Hutchinson P.J. (2017). Pathophysiology of chronic subdural haematoma: Inflammation, angiogenesis and implications for pharmacotherapy. J. Neuroinflamm..

[10] Ito H., Komai T., Yamamoto S. (1978). Fibrinolytic enzyme in the lining walls of chronic subdural hematoma. J. Neurosurg..

[11] Murakami H., Hirose Y., Sagoh M., Shimizu K., Kojima M., Gotoh K., Mine Y., Hayashi T., Kawase T. (2002). Why do chronic subdural hematomas continue to grow slowly and not coagulate? Role of thrombomodulin in the mechanism. J. Neurosurg..

[12] Hohenstein A., Erber R., Schilling L., Weigel R. (2005). Increased mRNA expression of VEGF within the hematoma and imbalance of angiopoietin-1 and -2 mRNA within the neomembranes of chronic subdural hematoma. J. Neurotrauma.

[13] Nakagawa T., Kodera T., Kubota T. (2000). Expression of matrix metalloproteinases in the chronic subdural haematoma membrane. Acta Neurochir..

[14] Dmitrieva N.I., Burg M.B. (2015). Elevated sodium and dehydration stimulate inflammatory signaling in endothelial cells and promote atherosclerosis. PLoS ONE.

[15] Park H.R., Lee K.S., Shim J.J., Yoon S.M., Bae H.G., Doh J.W. (2013). Multiple Densities of the Chronic Subdural Hematoma in CT Scans. J. Korean Neurosurg. Soc..

[16] Lacey J., Corbett J., Forni L., Hooper L., Hughes F., Minto G., Moss C., Price S., Whyte G., Woodcock T. (2019). A multidisciplinary consensus on dehydration: Definitions, diagnostic methods and clinical implications. Ann. Med..

[17] Eizenberg Y., Grossman E., Tanne D., Koton S. (2021). Admission Hydration Status and Ischemic Stroke Outcome-Experience from a National Registry of Hospitalized Stroke Patients. J. Clin. Med..

[18] Rowat A., Graham C., Dennis M. (2012). Dehydration in hospital-admitted stroke patients: Detection, frequency, and association. Stroke.

[19] Heinze G., Wallisch C., Dunkler D. (2018). Variable selection—A review and recommendations for the practicing statistician. Biom. J. Biom. Z..

[20] Stavrinou P., Katsigiannis S., Lee J.H., Hamisch C., Krischek B., Mpotsaris A., Timmer M., Goldbrunner R. (2017). Risk Factors for Chronic Subdural Hematoma Recurrence Identified Using Quantitative Computed Tomography Analysis of Hematoma Volume and Density. World Neurosurg..

[21] Jack A., O’Kelly C., McDougall C., Findlay J.M. (2015). Predicting recurrence after chronic subdural haematoma drainage. Can. J. Neurol. Sci..

[22] Yamamoto H., Hirashima Y., Hamada H., Hayashi N., Origasa H., Endo S. (2003). Independent predictors of recurrence of chronic subdural hematoma: Results of multivariate analysis performed using a logistic regression model. J. Neurosurg..

[23] Unterhofer C., Freyschlag C.F., Thome C., Ortler M. (2016). Opening the Internal Hematoma Membrane Does Not Alter the Recurrence Rate of Chronic Subdural Hematomas: A Prospective Randomized Trial. World Neurosurg..

[24] Majovsky M., Masopust V., Netuka D., Benes V. (2016). Flexible endoscope-assisted evacuation of chronic subdural hematomas. Acta Neurochir..

[25] Song D.H., Kim Y.S., Chun H.J., Yi H.J., Bak K.H., Ko Y., Oh S.J. (2014). The Predicting Factors for Recurrence of Chronic Subdural Hematoma Treated with Burr Hole and Drainage. Korean J. Neurotrauma.

[26] Fujitani S., Ishikawa O., Miura K., Takeda Y., Goto H., Maeda K. (2017). Factors predicting contralateral hematoma growth after unilateral drainage of bilateral chronic subdural hematoma. J. Neurosurg..

[27] Hani L., Vulcu S., Branca M., Fung C., Z’Graggen W.J., Murek M., Raabe A., Beck J., Schucht P. (2019). Subdural versus subgaleal drainage for chronic subdural hematomas: A post hoc analysis of the TOSCAN trial. J. Neurosurg..

[28] Bartley A., Jakola A.S., Tisell M. (2020). The influence of irrigation fluid temperature on recurrence in the evacuation of chronic subdural hematoma. Acta Neurochir..

[29] Leroy H.A., Aboukais R., Reyns N., Bourgeois P., Labreuche J., Duhamel A., Lejeune J.P. (2015). Predictors of functional outcomes and recurrence of chronic subdural hematomas. J. Clin. Neurosci..

[30] Borger V., Vatter H., Oszvald A., Marquardt G., Seifert V., Guresir E. (2012). Chronic subdural haematoma in elderly patients: A retrospective analysis of 322 patients between the ages of 65–94 years. Acta Neurochir..

[31] Gelabert-Gonzalez M., Iglesias-Pais M., Garcia-Allut A., Martinez-Rumbo R. (2005). Chronic subdural haematoma: Surgical treatment and outcome in 1000 cases. Clin. Neurol. Neurosurg..

[32] Uno M., Toi H., Hirai S. (2017). Chronic Subdural Hematoma in Elderly Patients: Is This Disease Benign?. Neurol. Med. Chir..

[33] Fotenos A.F., Mintun M.A., Snyder A.Z., Morris J.C., Buckner R.L. (2008). Brain volume decline in aging: Evidence for a relation between socioeconomic status, preclinical Alzheimer disease, and reserve. Arch. Neurol..

[34] Jang K.M., Choi H.H., Mun H.Y., Nam T.K., Park Y.S., Kwon J.T. (2020). Critical Depressed Brain Volume Influences the Recurrence of Chronic Subdural Hematoma after Surgical Evacuation. Sci. Rep..

[35] Nakamura K., Brown R.A., Araujo D., Narayanan S., Arnold D.L. (2014). Correlation between brain volume change and T2 relaxation time induced by dehydration and rehydration: Implications for monitoring atrophy in clinical studies. Neuroimage Clin..

[36] Elias S., Hoffman R., Saharov G., Brenner B., Nadir Y. (2016). Dehydration as a Possible Cause of Monthly Variation in the Incidence of Venous Thromboembolism. Clin. Appl. Thromb. Hemost..

[37] Hooper L., Bunn D.K., Downing A., Jimoh F.O., Groves J., Free C., Cowap V., Potter J.F., Hunter P.R., Shepstone L. (2016). Which Frail Older People Are Dehydrated? The UK DRIE Study. J. Gerontol. A Biol. Sci. Med. Sci..

[38] Teno J.M., Gozalo P., Mitchell S.L., Tyler D., Mor V. (2013). Survival after multiple hospitalizations for infections and dehydration in nursing home residents with advanced cognitive impairment. JAMA.

[39] Liu K., Pei L., Gao Y., Zhao L., Fang H., Bunda B., Fisher L., Wang Y., Li S., Li Y. (2019). Dehydration Status Predicts Short-Term and Long-Term Outcomes in Patients with Cerebral Venous Thrombosis. Neurocrit. Care.

[40] Montano N., Stifano V., Skrap B., Mazzucchi E. (2017). Management of residual subdural hematoma after burr-hole evacuation. The role of fluid therapy and review of the literature. J. Clin. Neurosci..

[41] Janowski M., Kunert P. (2012). Intravenous fluid administration may improve post-operative course of patients with chronic subdural hematoma: A retrospective study. PLoS ONE.

